# Optimizing gene prioritization for clinical diagnosis of metabolic genetic disorders

**DOI:** 10.1371/journal.pone.0331038

**Published:** 2025-08-29

**Authors:** Beatriz Helena Dantas Rodrigues de Albuquerque, Daniel Carlos Ferreira Lanza

**Affiliations:** Laboratory of Applied Molecular Biology (LAPLIC), Department of Biochemistry, Federal University of Rio Grande do Norte, Natal, Rio Grande do Norte, Brazil; Shaheed Rajaei Cardiovascular Medical and Research Center: Rajaie Cardiovascular Medical and Research Center, IRAN, ISLAMIC REPUBLIC OF

## Abstract

The expansion of next-generation sequencing has generated vast genomic datasets, but translating this information into clinically actionable tools for inherited metabolic disorders (IMDs) remains challenging. In this study, we systematically mapped gene–phenotype associations in IMDs using curated data from OMIM, ClinVar, Orphanet, and the Genetic Testing Registry (GTR). From 372 OMIM entries, we identified 228 genes definitively associated with metabolic diseases (GAMD). These genes displayed uneven chromosomal distribution, wide variability in pathogenic variant load, and strong clustering of phenotypes, particularly among amino acid metabolism disorders. Autosomal recessive inheritance was predominant. Integrating variant pathogenicity, phenotype prevalence, and diagnostic test availability, we designed two evidence-based diagnostic panels. The Subnotification Panel highlights under-tested but clinically relevant genes linked to more prevalent IMDs, aiming to address diagnostic underrepresentation. The Initial Screening Panel prioritizes genes with a high proportion of pathogenic variants, broad test accessibility, and strong clinical relevance, offering an efficient tool for first-line diagnostics. By bridging the gap between large-scale genomic information and precision clinical application, these panels provide a scalable and strategic framework to enhance diagnostic accuracy, support early intervention, and improve equity in the management of metabolic diseases.

## Introduction

Inherited metabolic disorders (IMDs) comprise a heterogeneous group of rare genetic diseases that, although individually uncommon, have a significant cumulative incidence [1–2]. Approximately 1,450 disorders have been cataloged to date in the International Classification of Inherited Metabolic Disorders (ICIMD) [3]. Most IMDs follow an autosomal recessive inheritance pattern and typically manifest early in life, often during the neonatal period, although some may only present later, with subtle symptoms during adolescence or adulthood [4].

The availability of established therapies for several IMDs has justified their inclusion in neonatal screening programs worldwide [5]. Nevertheless, the remarkable clinical and genetic heterogeneity of IMDs imposes significant diagnostic challenges, especially when early intervention is critical [2,4]. Overlapping clinical features and variability in age of onset further complicate the accurate identification of these conditions.

Genetic testing has emerged as a pivotal tool to overcome diagnostic barriers, improving the accuracy of diagnosis and guiding prognosis, therapeutic strategies, and genetic counseling [4,6]. Advances in genomics have driven a rapid expansion of known disease-related variants and associated genes [7], revealing that similar phenotypes can arise from diverse underlying molecular mechanisms. Technologies such as whole-genome sequencing (WGS), whole-exome sequencing (WES), and targeted panels have substantially improved IMD detection, particularly when integrated with clinical data within the framework of precision medicine [7–8].

Initially, targeted panels focusing on specific metabolic pathways offered a major leap forward in diagnostics by increasing detection rates while minimizing complexity. However, the continuous growth of genomic information has outpaced traditional panel design, generating an overwhelming volume of data. Many newly identified targets remain rare or poorly characterized, with undefined genotype–phenotype correlations, complicating variant interpretation [9–10]. As a result, the diagnostic challenge has shifted from accessing sufficient genomic information to the effective triage and interpretation of clinically relevant findings.

The current landscape demands the strategic refinement of genetic testing: prioritizing markers with robust clinical utility while minimizing noise from variants of uncertain significance (VUS) [11–12]. In this context, carefully curated, purpose-driven diagnostic panels emerge as essential tools to navigate the increasing complexity and deliver actionable insights more efficiently.

In this study, we present a systematic characterization of genes and variants associated with IMDs, mapping gene–phenotype relationships using curated databases. Based on this analysis, we propose two targeted diagnostic panels: one addressing the underrepresentation of clinically relevant yet under-tested conditions, and another prioritizing high-yield genes with greater pathogenicity burden and broad clinical applicability. These resources aim to optimize diagnostic workflows, enhance early detection, and promote the integration of precision medicine into the management of metabolic diseases.

## Materials and methods

### Data collection and gene identification

To identify genes associated with inherited metabolic disorders (IMDs), we queried the Online Mendelian Inheritance in Man (OMIM®; https://www.omim.org/) database [13] in January 2023. The search strategy combined multiple keywords, including “metabolic diseases”, “metabolic disorders”, “inborn errors of metabolism”, “inborn error of metabolism”, “inherited metabolic diseases”, and “inherited metabolic disorders”. Only OMIM entries with documented phenotypes listed in the OMIM Gene Map were included for further analysis.

### Variant data extraction

For each identified gene, we retrieved the total number of reported genetic variants from the ClinVar database (https://www.ncbi.nlm.nih.gov/clinvar/). Special attention was given to variants classified as pathogenic. Variant counts were extracted systematically to quantify the mutational burden associated with each gene.

### Genetic testing availability

The total number of available genetic tests per gene was recorded using the Genetic Testing Registry (GTR®; https://www.ncbi.nlm.nih.gov/gtr/), a publicly accessible database maintained by the National Institutes of Health (NIH). Data collection from the GTR focused on capturing the extent of clinical testing availability for each gene at the time of analysis.

### Chromosomal mapping

Chromosomal locations of the identified genes were visualized using R software (version 4.3.0), employing the karyoploteR package [14]. This allowed graphical representation of the genomic distribution of genes associated with IMDs.

### Phenotype categorization

Phenotype classification was guided by two main resources: the International Classification of Inherited Metabolic Disorders (ICIMD) [3] and IEMbase (version 2.0.0; www.iembase.org), an online platform dedicated to the classification of inborn errors of metabolism. Each gene was mapped to its respective metabolic phenotype category based on these curated frameworks.

### Prevalence estimation

Phenotypic burden was assessed using the point prevalence metric, representing the total number of affected individuals within a given population at a specific time [15]. Prevalence data were retrieved from the Orphanet database (www.orpha.net). All prevalence estimates, pathogenic variant counts, and genetic testing availability metrics reflect data collected as of January 2025.

## Results

### Identification of genes associated with metabolic disorders

A total of 372 OMIM entries related to metabolic disorders were identified. Among these, 317 entries were mapped to specific genes, and 294 contained detailed phenotype information. Ultimately, 228 genes were definitively associated with inherited metabolic disorders and classified as Genes Associated with Metabolic Disorders (GAMD), forming the basis for all subsequent analyses (S1 Table).

### Genomic distribution and variant profiling

**Fig 1:**
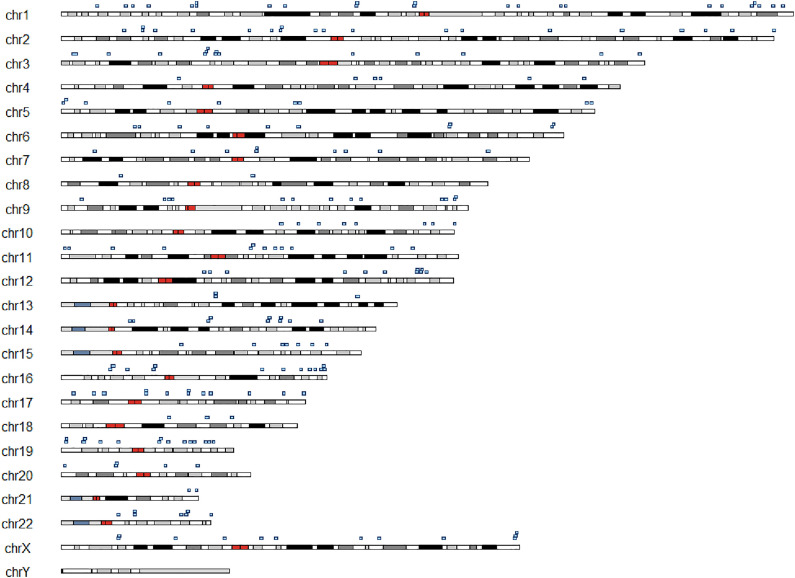
Chromosomal distribution of genes associated with metabolic disorders (GADM). Idiogram showing the genomic location of 228 genes associated with inherited metabolic disorders (GADM). Blue rectangles mark the position of each gene on the corresponding chromosome. Genes are distributed across all chromosomes except the Y chromosome, with notable clustering on chromosomes 1, 2, and 19.

The 228 GAMD were distributed across all human chromosomes except the Y chromosome (Fig 1). Chromosomes 1, 2, and 19 harbored the highest number of associated genes, with 24, 20, and 15 genes, respectively.

Variant analysis using ClinVar revealed a mean of 587.62 (± 564.94) total variants per gene, with an average of 95.94 (± 104.94) pathogenic variants. *APOB* exhibited the highest variant count (n = 3,977), whereas *NDUFC2* and *COX16* had the lowest counts (22 and 25 variants, respectively). *ATP7B* had the greatest number of pathogenic variants (n = 557), while *COX14* and *HAL* presented the fewest (n = 5 each).

**Fig 2:**
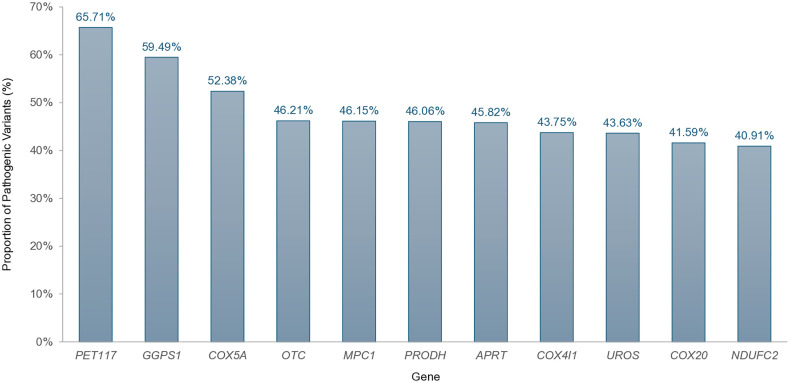
GADM genes with the highest proportion of pathogenic variants. Bar plot highlighting the subset of genes associated with metabolic disorders that present ≥40% of their ClinVar-reported variants classified as pathogenic. This distribution emphasizes the heterogeneity in diagnostic informativeness across different GADM.

Importantly, 11 genes showed ≥40% of their variants classified as pathogenic (Fig 2), while 56 genes—including *APOB*—had less than 10% pathogenic variants, highlighting the considerable heterogeneity in clinical relevance among GAMD (S2 Table).

### Phenotypic spectrum and inheritance patterns

The 228 GAMD were collectively linked to 289 distinct phenotypes. According to the ICIMD framework, the most frequent category was “disorders of amino acid metabolism” (20.41%, 59/289), followed by “nuclear-encoded disorders of oxidative phosphorylation” (35/289), “disorders of vitamin and cofactor metabolism” (30/289), and both “disorders of carbohydrate metabolism” and “nucleotide/nucleic acid metabolism” (21/289 each) (Table 1).

**Table 1:** Phenotypic categories associated with the 228 GAMD genes and their most prevalent subcategories.

Category	Number of phenotypes	Number of genes	Most prevalent subcategory
Disorders of amino acid metabolism	59	53	Organic acidurias
Nuclear-encoded disorders of oxidative phosphorylation	35	26	Disorders of complex IV subunits and assembly factors
Disorders of vitamin and cofactor metabolism	30	26	Disorders of cobalamin metabolism
Disorders of carbohydrate metabolism	21	18	Disorders of glycogen metabolism
Disorders of nucleobase, nucleotide and nucleic acid metabolism	21	12	Disorders of ectonucleotide and nucleic acid metabolism
Disorders of lipid metabolism	18	14	Disorders of bile acid metabolism
Congenital disorders of glycosylation	14	12	Disorders of Golgi homeostasis
Disorders of complex molecule degradation	12	8	Disorders of sphingolipid degradation
Disorders of fatty acid and ketone body metabolism	11	10	Disorders of mitochondrial fatty acid oxidation
Disorders of organelle biogenesis, dynamics and interactions	10	6	Peroxisomal biogenesis disorders
Disorders of lipoprotein metabolism	9	5	Mixed hyperlipidemias
Endocrine metabolic disorders	9	3	Disorders of insulin metabolism
Disorders of tetrapyrrole metabolism	7	5	Disorders of heme synthesis and porphyrias
Disorders of energy substrate metabolism	6	5	Disorders of pyruvate metabolism
Disorders of peptide and amine metabolism	7	6	Disorders of glutathione metabolism and disorders of methylamine metabolism
Other disorders of mitochondrial function	5	4	Disorders of mitochondrial protein quality control
Disorders of metabolite repair/proofreading	3	3	Disorders of mitochondrial metabolite repair
Disorders of mitochondrial cofactor biosynthesis	3	3	Disorders of lipoic acid and iron-sulfur metabolism
Disorders of trace elements and metals	3	3	Disorders of manganese metabolism, disorders of iron metabolism and disorders of copper metabolism
Miscellaneous disorders of intermediary metabolism	2	2	Disorders of glyoxylate and oxalate metabolism and unassigned disorders of intermediary metabolism
Neurotransmitter disorders	2	2	Glycine neurotransmitter disorders and gamma-aminobutyric acid neurotransmitter disorders
Disorders of mitochondrial DNA maintenance and replication	1	1	Disorders of mitochondrial nucleotide pool maintenance
Disorders of mitochondrial gene expression	1	1	Disorders of mitochondrial aminoacyl-tRNA synthetases
Total	289	228	

Categories of the 289 distinct phenotypes associated with the 228 GAMD genes. For each phenotypic category, the number of related phenotypes and genes is shown, along with the most prevalent subcategory within each group.

Inheritance pattern analysis revealed that autosomal recessive transmission predominated, accounting for 85.86% (249/290) of phenotypes with known inheritance modes.

### Prevalence and diagnostic visibility

Prevalence data retrieved from Orphanet showed a wide range of occurrence among the 289 phenotypes. Of these, 123 were classified as extremely rare (<1/1,000,000), 24 fell within 1–9 per 1,000,000, 21 within 1–9 per 100,000, and 6 within 1–5 per 10,000. Prevalence data were unavailable for 115 phenotypes (S3 Table)

The most prevalent phenotypes (1–5 per 10,000) included dilated cardiomyopathy, MODY2, MODY10, cystinuria, hyperlipoproteinemia type III, and Fabry disease. These conditions were associated with genes exhibiting varying pathogenicity ratios, such as *SDHA* (10.80%) and *GLA* (39.88%) (S2 Table).

Nineteen genes were linked to phenotypes with a prevalence between 1–9 per 100,000, with many demonstrating a high proportion of pathogenic variants, including *OTC* (46.21%), *APRT* (45.81%), and *ATP7B* (17.46%) (S2 Table).

Analysis of GTR records revealed substantial disparities in genetic test availability. *FH* had the highest number of registered tests (n = 274), followed by *SDHD*, *GLA*, *SDHA*, *PEX1*, *TAFAZZIN*, *ATP7B*, *ALPL*, and *ACADM*, each with over 200 available tests. Conversely, genes such as *PHYKPL*, *LDHD*, *SLC28A1*, and *COX16* had fewer than five tests each, indicating significant underrepresentation in clinical diagnostics (S4 Table).

### Integrative prioritization for diagnostic panel design

**Fig 3:**
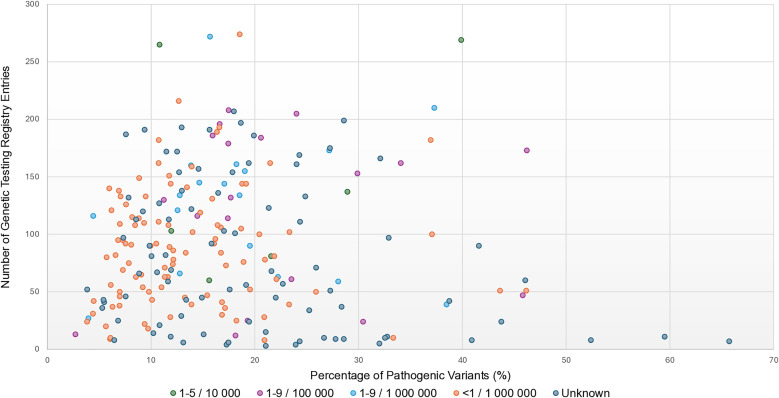
Prioritization of GADM based on pathogenic variant ratio, test availability, and phenotype prevalence. Scatterplot showing the integrated evaluation of each GADM gene considering (i) the proportion of variants classified as pathogenic, (ii) the number of registered clinical tests in the Genetic Testing Registry (GTR), and (iii) the prevalence of associated phenotypes as reported in Orphanet. Genes positioned in the upper right quadrant represent candidates with both diagnostic maturity and clinical relevance.

By combining data on variant pathogenicity, phenotype prevalence, and test availability (Fig 3), we developed a strategic prioritization of GAMD for clinical application, culminating in the proposal of two complementary diagnostic panels.

### Subnotification panel: Addressing underreported but clinically relevant genes

Recent analyses indicate a decline in novel gene discoveries since 2013, with a shift toward new phenotypes associated with known genes—38% of updates in OMIM and 43% in Orphanet [16]. This trend highlights a diagnostic bottleneck not in gene discovery, but in the underutilization of existing genes.

To bridge this gap, we identified GAMD associated with relatively prevalent phenotypes (1–9/100,000 or 1–5/10,000) that remain underrepresented in genetic testing (defined as having fewer than the GAMD mean of 91 tests per gene). These genes were compiled into a Subnotification Panel (Table 2), targeting conditions likely to be clinically missed due to testing gaps.

**Table 2:** Subnotification panel.

Gene	GTR	Prevalence	Phenotype	Inheritance	Category
*INS*	81	1-5/ 10 000	Permanent neonatal diabetes mellitus 4; hyperproinsulinemia, maturity-onset diabetes of the young type 10	AR; AD	Endocrine metabolic disorders
*SLC6A19*	61	1-9/ 100 000	Hartnup disorder; iminoglycinuria	AR; DR	Disorders of amino acid metabolism
*SLC7A9*	60	1-5/ 10 000	Cystinuria	AR; AD	Disorders of amino acid metabolism
*APRT*	47	1-9/ 100 000	Adenine phosphoribosyltransferase deficiency	AR	Disorders of nucleobase, nucleotide and nucleic acid metabolism
*CTH*	25	1-9/ 100 000	Cystathioninuria	AR	Disorders of amino acid metabolism
*CAT*	24	1-9/ 100 000	Acatalasemia	AR	Miscellaneous disorders of intermediary metabolism
*HAL*	13	1-9/ 100 000	Histidinemia	AR; AD	Disorders of amino acid metabolism
*SARDH*	12	1-9/ 100 000	Sarcosinemia	AR	Disorders of peptide and amine metabolism

AR, autosomal recessive; AD, autosomal dominant.

Most genes included in this panel are involved in amino acid metabolism, providing a phenotypically cohesive group that supports focused diagnostic strategies and may offer higher yield compared to untargeted WES/WGS approaches [17].

### Initial screening panel: high pathogenic load and diagnostic maturity

A second panel was developed to optimize first-line genetic screening by selecting GAMD that met three criteria:

-  ≥ 10% of variants classified as pathogenic;
- Test availability above the GAMD mean based on GTR records;
- Association with phenotypes of moderate to high prevalence.

This Initial Screening Panel (Table 3) features genes that are both biologically and diagnostically mature. Their inclusion promotes high-confidence clinical interpretation, minimizes ambiguity from variants of uncertain significance, and supports efficient deployment in neonatal and early-onset diagnostic workflows.

**Table 3:** Initial screening panel.

Gene	Percentage of Pathogenic Variants (%)	GTR	Prevalence	Phenotype	Inheritance	Category
*OTC*	46. 21	173	1-9/ 100 000	Ornithine transcarbamylase deficiency	XL	Disorders of amino acid metabolism
*GLA*	39.88	269	1-5/ 10 000	Fabry disease; cardiac variant of Fabry disease	XL	Disorders of complex molecule degradation
*AR*	34.06	162	1-9/ 100 000	Androgen insensitivity; androgen insensitivity, partial, with or without breast cancer; spinal and bulbar muscular atrophy of Kennedy	XL	Endocrine metabolic disorders
*PAH*	29.87	153	1-9/ 100 000	Phenylketonuria; non-PKU mild hyperphenylalaninemia	AR	Disorders of amino acid metabolism
*GCK*	28.91	137	1-5/ 10 000	Permanent neonatal diabetes mellitus-1; familial hyperinsulinemic hypoglycemia-3; maturity-onset diabetes of the young type 2	AR; AD	Disorders of carbohydrate metabolism
*ACADM*	24.00	205	1-9/ 100 000	Deficiency of medium-chain acyl-CoA dehydrogenase	AR	Disorders of fatty acid and ketone body metabolism
*BTD*	20.59	184	1-9/ 100 000	Biotinidase deficiency	AR	Disorders of vitamin and cofactor metabolism
*ASS1*	17.67	132	1-9/ 100 000	Citrullinemia	AR	Disorders of amino acid metabolism
*ATP7B*	17.46	208	1-9/ 100 000	Wilson disease	AR	Disorders of trace elements and metals
*GALC*	17.43	179	1-9/ 100 000	Krabbe disease	AR	Disorders of complex molecule degradation
*ABCC6*	17.39	114	1-9/ 100 000	Generalized arterial calcification of infancy-2; Pseudoxanthoma elasticum; Pseudoxanthoma elasticum, forme fruste	AR; AD	Disorders of nucleobase, nucleotide and nucleic acid metabolism
*CBS*	16.60	196	1-9/ 100 000	Homocystinuria, B6-responsive and nonresponsive types; Thrombosis, hyperhomocysteinemic	AR	Disorders of amino acid metabolism
*GNE*	15.91	186	1-9/ 100 000	Nonaka myopathy; Sialuria	AR; AD	Congenital disorders of glycosylation
*ASL*	14.46	116	1-9/ 100 000	Argininosuccinic aciduria	AR	Disorders of amino acid metabolism
*APOE*	11.94	103	1-5/ 10 000	Hyperlipoproteinemia, type III; Lipoprotein glomerulopathy; Sea-blue histiocyte disease	AR	Disorders of lipoprotein metabolism
*IVD*	11.22	130	1-9/ 100 000	Isovaleric acidemia	AR	Disorders of amino acid metabolism
*SDHA*	10.80	265	1-5/ 10 000	Cardiomyopathy, dilated, 1GG; mitochondrial complex II deficiency nuclear type 1; Paragangliomas 5	AR; AD	Nuclear-encoded disorders of oxidative phosphorylation

AR, autosomal recessive; AD, autosomal dominant; XL, X-linked.

## Discussion

### Genetic landscape of inherited metabolic disorders: Complexity and diagnostic potential

This study provides a systematic and integrative characterization of 228 genes associated with inherited metabolic disorders (GAMD), combining curated data on variant burden, phenotypic associations, prevalence, and diagnostic availability. Unlike previous reports that focused on specific subgroups or clinical presentations, our approach offers a broad, structured view of the genetic architecture of metabolic diseases, enabling evidence-based panel design.

Chromosomal mapping revealed a non-random distribution of GAMD, with enrichment on chromosomes 1, 2, and 19, consistent with previous studies suggesting the clustering of disease-associated genes in specific genomic regions [18]. Although such distributions may partly reflect gene density, they also suggest functional co-localization and coordinated regulation that merit further investigation in the context of metabolic disease susceptibility.

Variant analysis reinforced the complexity inherent to pathogenic interpretation. Despite the high number of variants per gene, only a minority of GAMD exhibited a high proportion of pathogenic variants. For example, *APOB* contained nearly 4,000 variants but had only 4.42% classified as pathogenic, whereas *PET117* had a much smaller total variant count but over 65% pathogenicity. These disparities illustrate underlying biological mechanisms, such as mutational robustness and functional constraint, emphasizing the importance of gene-level contextualization in variant interpretation [19–20] — a foundational principle reflected in the diagnostic panels proposed herein.

### Prevalence, data gaps, and underutilization of diagnostic resources

Prevalence data obtained from Orphanet revealed that approximately 40% of GAMD-linked conditions lacked defined prevalence estimates, underscoring both the extreme rarity of many IMDs and persistent systemic underreporting [21]. Even among more common phenotypes, gaps in clinical recognition and test utilization persist.

Diagnostic test availability demonstrated striking disparities. While some genes, such as *FH*, were extensively tested and well-represented in clinical workflows, others like *PHYKPL* and *SLC28A1* had fewer than five registered tests, despite association with recognizable metabolic phenotypes. This mismatch highlights critical blind spots in current diagnostic strategies, where clinically relevant but underexplored genes may remain undetected.

By quantifying these discrepancies, our study provides empirical support for a more equitable, clinically driven distribution of genetic testing resources, prioritizing clinical utility over commercial considerations.

### Translational output: Targeted panel design for precision diagnostics

#### Subnotification panel: Bridging the gap between relevance and recognition.

One of the key innovations of this work is the development of the Subnotification Panel, targeting underrepresented but clinically significant genes. By integrating phenotypic prevalence, variant pathogenicity, and test availability, we identified a subset of GAMD genes that are both relevant and currently underutilized in clinical diagnostics.

Incorporating these genes into routine testing workflows has the potential to uncover missed diagnoses, accelerate therapeutic interventions, and enhance health equity, particularly in under-resourced settings. The biological cohesion of this panel—dominated by genes involved in amino acid metabolism—further increases its diagnostic yield and interpretability compared to broader untargeted approaches [17].

#### Initial screening panel: Prioritizing diagnostic power.

In parallel, we developed the Initial Screening Panel, prioritizing GAMD genes based on three converging criteria: a high proportion of pathogenic variants (≥10%), broad availability of genetic testing, and association with phenotypes of moderate to high prevalence.

This strategy emphasizes genes that are biologically and diagnostically mature, enabling rapid, cost-effective, and high-confidence clinical screening. The panel minimizes analytical ambiguity, reducing the burden of variants of uncertain significance and facilitating streamlined clinical decision-making in neonatal and early-onset diagnostic contexts.

### Study limitations

This study presents some inherent limitations. First, our analyses were intentionally based on publicly available, standardized databases (OMIM, ClinVar, Orphanet, and GTR), selected to ensure transparency and reproducibility. While widely accepted, these resources have known limitations, including curation delays, regional biases, and incomplete coverage—particularly of variants of uncertain significance (VUS) and rare conditions. In the context of inherited metabolic disorders (IMDs), pathogenic variants may still be listed as VUS or absent altogether from these repositories [22–23].

ClinVar, for instance, depends on voluntary submissions and emphasizes variants with clinical or experimental support, often omitting observational or unpublished findings [24]. Currently, over 40% of its variants are classified as VUS or have conflicting interpretations [23]. Despite quality controls, inconsistencies remain common due to varying interpretations across contributors [25]. Many VUS may later be reclassified as new evidence emerges [22].

Although literature-based curation could improve classification precision, we deliberately avoided this to maintain methodological consistency, reduce bias, and support reproducibility. Manual review from dispersed sources would limit replicability. Instead, our framework is openly accessible and adaptable, allowing future updates as databases evolve. Expert-curated evidence may be incorporated in later versions, provided it aligns with scalable and reproducible practices.

Second, prevalence data are unavailable for many rare phenotypes, limiting prioritization. Third, available prevalence estimates are not stratified by ancestry or region, which may affect local applicability. Incorporating regional data is a valuable future direction.

Fourth, GTR data reflect primarily U.S.-based test availability and may not fully represent global practices. Still, it remains the most comprehensive and standardized resource of its kind.

In summary, while these limitations are expected in large-scale analyses using open genomic data, they were considered in our study design and do not compromise its validity. They reinforce the importance of building adaptable, reproducible frameworks that can be refined as evidence and clinical contexts evolve.

### Broader implications and future perspectives

The global burden of rare diseases remains characterized by delayed diagnoses, limited testing access, and significant disparities across different populations [26]. By proposing a structured and data-driven prioritization framework, this study contributes to a more inclusive model of precision medicine, identifying overlooked targets and optimizing diagnostic workflows.

Importantly, our findings also highlight current limitations in genomic databases, particularly the overrepresentation of European ancestry and the underrepresentation of pathogenic variants from other populations [27]. Expanding genomic reference datasets to include more diverse populations, and integrating complementary omics approaches such as transcriptomics and metabolomics, will be critical to fully realizing the promise of genomic medicine in the management of metabolic disorders.

## Conclusion

This study provides a comprehensive and integrative overview of genes associated with inherited metabolic disorders, combining variant burden, phenotypic prevalence, and diagnostic accessibility to inform precision diagnostics. By identifying under-tested yet clinically relevant genes and prioritizing those with high pathogenic potential, we developed two complementary diagnostic panels designed to address critical gaps in clinical genomics. These panels offer scalable, evidence-based solutions to enhance diagnostic accuracy, accelerate time to diagnosis, and support early intervention strategies in metabolic medicine. By delivering a clear and reproducible framework for gene prioritization, this work contributes to the transition from exploratory sequencing toward more strategic, resource-conscious diagnostics, ultimately advancing the clinical management of rare diseases.

## Supporting information

S1 Table Overview of all 228 GAMD genes. (XLSX)

S2 Table Proportion of pathogenic variants per gene. (XLSX)

S3 Table Prevalence of the 289 phenotypes. (XLSX)

S4 Table Number of available tests per gene in the GTR. (XLSX)
